# Total nitrogen is the main soil property associated with soil fungal community in karst rocky desertification regions in southwest China

**DOI:** 10.1038/s41598-021-89448-1

**Published:** 2021-05-24

**Authors:** Daihua Qi, Xuwen Wieneke, Peipei Xue, Li He, Udaya DeSilva

**Affiliations:** 1 Key Laboratory of Eco-Environments in Three Gorges Reservoir Region (Ministry of Education), Chongqing Key Laboratory of Plant Ecology and Resources Research in Three Gorges Reservoir Region, School of Life Sciences, Southwest University, Chongqing, China; 2 Department of Animal Science, Oklahoma State University, Stillwater, OK USA; 3 The Forestry Academy of Chongqing, Chongqing, China; 4 The Forestry Academy of Sichuan, Sichuan, China

**Keywords:** Soil microbiology, Environmental impact

## Abstract

Karst rocky desertification (KRD) is a type of land deterioration, resulting in the degraded soil and a delicate ecosystem. Previous studies focused on the influence of KRD on the animals and plants, the impact of KRD on microorganisms, especially soil fungi remains to be discovered. This study reveals the change in the soil fungal community in response to KRD progression in southwest China. Illumina HiSeq was used to survey the soil fungal community. Results showed that the soil fungal community in the severe KRD (SKRD) was noticeably different from that in other KRD areas. Statistical analyses suggested that soil TN was the primary factor associated with the fungal community, followed by pH. Phylum Ascomycota was significantly abundant in non-degraded soils; whereas Basidiomycota predominated in SKRD. The ratio of Ascomycota/Basidiomycota significantly decreased along with KRD progression, which might be used as an indicator of KRD severity. Phylum Basidiomycota was sensitive to changes in all the soil properties but AP. Genus *Sebacina* might have the potential to promote vegetation and land restoration in KRD areas. This study fills a gap of knowledge on changes in soil fungal communities in accordance with KRD progression.

## Introduction

Karst is a topography resulting from the dissolution of soluble rocks, e.g., limestone, dolomite, and gypsum. Karst is a natural landscape, found in some well-known regions including the European Mediterranean, the Dinaric karst regions of the Balkan Peninsula, and in Southwest China. The ecosystem in the karst region is fragile; therefore, the land in the karst area is prone to deterioration, resulting in the karst rocky desertification (KRD)^1^. The progression of Karst rocky desertification (KRD) leads to soil content loss, bedrock exposure, vegetation community disruption, and decline of soil productivity^2^. The karst landscape largely exists in Wushan County, Chongqing (30°46′–31°28′ N, 109°33′–110°11′ E), with over 70% of soils (2066 km2) prone to soil deterioration^3^. The large amount of KRD land and its low productivity pose challenges to the local economy and global ecosystem. KRD restoration has been one of the key projects in China^4^. Understanding the interaction of KRD with the ecosystem is the first step to KRD rehabilitation.

The impact of KRD on the above-ground communities, e.g., vegetation and animals, has been well-characterized. The progression of KRD discomposes the vegetation, in terms of complexity, diversity, and richness^5^. Changes in vegetation, in turn, affect the soil properties and microbiome^6,7^. The next generation sequencing (NGS) technologies are powerful tools to study the interaction of KRD with the underground community, soil microbiome, in a high resolution. Some studies reveal changes in soil bacterial community and core microbiome in response to KRD progression, vegetation type, and vegetation restoration^8–10^. Few studies, however, aim to elucidate the relationship of soil fungal community and core microbiome with KRD progression. A core microbiome is defined as “two or more microbial groups in a shared habitat”^11,12^. Identifying the core microbiome in both healthy and deteriorated land is critical for understanding the microbial ecology and predicting the microbial responses to the disturbance, in this case KRD progression. Furthermore, understanding the core microbiome composition provides an insight on the essential functions of microbiome in KRD regions; thereby outlining the microbiome management for KRD restoration.

Soil microorganisms are highly sensitive to soil physicochemical properties^13,14^. Soil fungi, one of the major components of soil microorganisms, have a large impact on the soil ecology acting as soil organic matter (SOM) decomposers and nutrient cycling assistants^15^. There are numerous fungi that break down SOM in the terrestrial ecosystem, contributing to soil nutrient cycling. To name a few, Ascomycota, one of the largest phyla, play an important role in decaying and lichenization^16^. Agaricomycotina (phylum Basidiomycota), are known for the heterologous aromatic polymer (e.g., lignin) decomposition^17^. Phylum Glomeromycota is widespread in terrestrial ecosystems, with abilities to facilitate nutrient recycling in soil and benefit host plants^18^.

The fungal community is also highly dynamic and interactive to its surroundings, e.g., vegetation, soil type and physicochemical properties, altitude, and climate^19–23^. Indeed, studies reveal a cooperative effect of organic matter and pH on fungi, changing the richness and the distribution of decomposer fungi in the community^24,25^. Change in the fungal community, in turn, has influences on the decomposition rate, thereby alternating the intensity of soil exploitation and nutrient availability^26^. In general, primary plant type, soil pH, and soil nutrient contents cooperatively affect the soil fungal community^27–31^. Little is known, however, on change in the soil fungal community in response to the progression of KRD. Moreover, few studies investigated the relationship of soil properties with the soil fungal community. Revealing the essential environmental factor affecting the soil fungal community will help to preserve the healthy soil microbiome in the KRD region.

In this study, soil fungal communities were characterized by deep sequencing the internal transcribed spacer 1 (ITS1) region via Illumina HiSeq platform. Soil properties, pH, soil organic matter (SOM), total and available nitrogen (TN and AN), total and available phosphorus (TP and AP), and total and available potassium (TK and AK) were measured in KRD regions. The goals were 1) to reveal the change in soil fungal community and identify the core microbiome in KRD progression; 2) to determine which soil property is the major factor that significantly correlates with soil fungal community; and 3) to reveal the relationship of soil properties with core taxa.

## Results

A total of 1,614,053 raw paired-end sequencing reads were obtained from the sequencing. The data trimming and random subsampling left 920,702 qualified reads, with an average of 76,725 ± 30,336 reads per sample.

A total of 155,774 OTUs were identified, with 8 phyla (Ascomycota, Basidiomycota, Glomeromycota, Mucoromycota, Chytridiomycota, Neocallimastigomycota, Zoopagomycota, and Blastocladiomycota). All the qualified reads were further classified into 23 classes, 82 orders, 184 families, and 352 genera. Detailed OTU information is shown in Supplementary Table 1.

**Figure 1:**
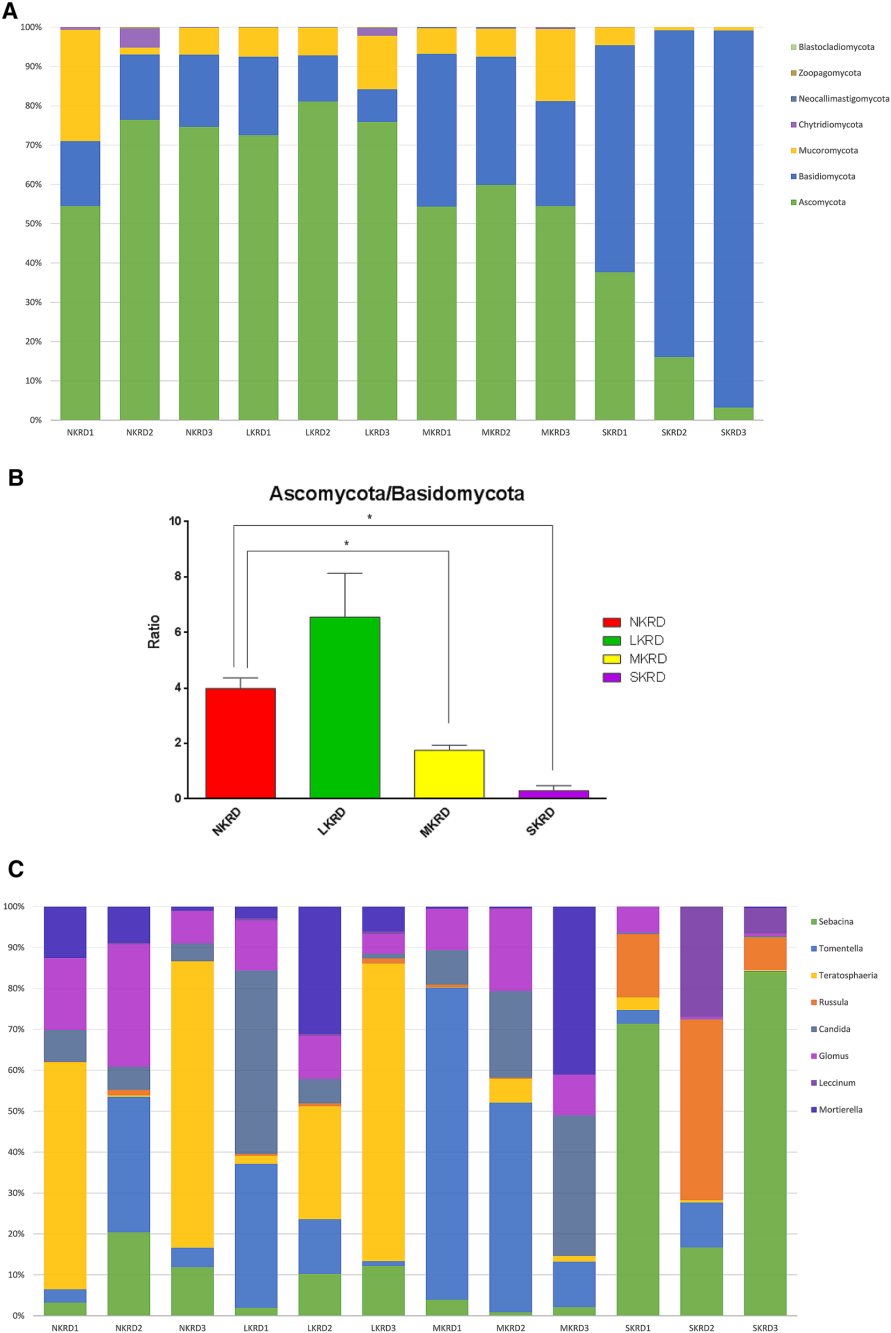
Relative abundance of fungi at phylum level (**A**), ascomycota/basidiomycota ratio (**B**), and core genera (**C**). The Tukey's honest significant difference (HSD) test was used to determine the significance, *P < 0.05. No KRD (NKRD), latent KRD (LKRD), moderate KRD (MKRD), and severe KRD (SKRD).

The relative abundance of phyla is shown in Fig. 1A. Ascomycota and Basidiomycota were two of the most predominating phyla, with 52.94% and 37.29% of relative abundance among all the OTUs. Mucoromycota were the third most abundant phylum, taking 9.27% of the population, followed by Chytridiomycota (0.41%), Neocallimastigomycota (0.07%), Zoopagomycota (0.03%), and Blastocladiomycota (0.02%). The core microbiome was defined as taxa that existed in all the samples and took at least 1% of the relative abundance within a sample. Under such criteria, phyla Ascomycota, Basidiomycota, and Mucoromycota were the core microbiome in this study. Ascomycota claimed the predominance in N- and L-KRD regions, with an average of 71.82% of the reads. The fungal community in SKRD areas was predominated by Basidiomycota. The amount of Ascomycota sharply decreased and was outnumbered by Basidiomycota in SKRD. The ratio of Ascomycota/Basidiomycota significantly changed along with KRD progression (Fig. 1B).

Twenty-two genera accounted for more than 1% of the population, namely *Sebacina* (18.46%), *Tomentella* (8.03%), *Teratosphaeria* (7.57%), *Russula* (7.46%), *Candida* (5.03%), *Glomus* (4.28%), *Leccinum* (4.02%), *Mortierella* (3.91%), *Daedalea* (3.36%), *Cookeina* (2.63%), *Tricholoma* (2.21%), *Tapinella* (2.26%), *Clavaria* (2.00%), *Cryptococcus* (1.54%), *Olpidium* (1.44%), *Oidiodendron* (1.28%), *Cryptosporiopsis* (1.24%), *Capnobotryella* (1.13%), *Pluteus* (1.05%), and *Epulorhiza* (1.05%). The core genera were *Sebacina, Tomentella, Teratosphaeria, Russula, Candida, Glomus, Leccinum,* and *Mortierella*. The relative abundance of core genera is shown in Fig. 1C. Many of these genera had potential functions involved with decomposition, nutrient cycling, and mineral weathering in soil, which were associated with changes in soil properties.

**Figure 2:**
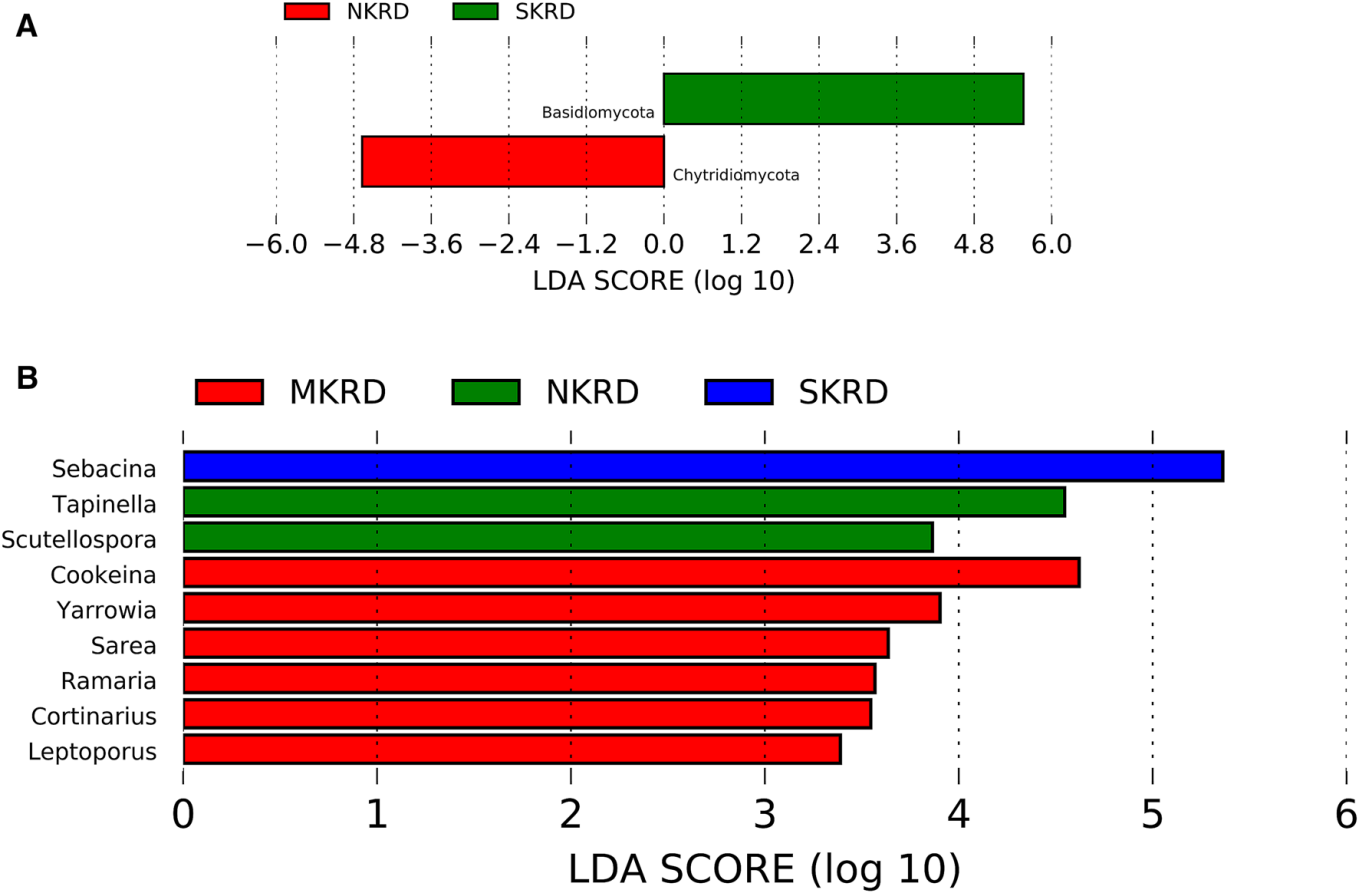
The Linear discriminant analysis (LDA) Effect Size (LEfSe) analysis was used to identify phylum (**A**) and genus (**B**) that respond significantly to karst rocky desertification progression. Relative abundance is significant when *P* < 0.05, logarithmic LDA score ≥ 2.0. No KRD (NKRD), Latent KRD (LKRD), Medium KRD (MKRD), and severe KRD (SKRD).

Lefse analysis was used to identify taxa that significantly responded to KRD progression. Phylum Chytridiomycota significantly decreased in NKRD; whereas Basidiomycota significantly increased in SKRD areas (Fig. 2A). The changes of Basidiomycota and Chytridiomycota in each KRD region are shown in Supplementary Fig. 1. Genera that significantly predominated in each KRD region were determined via Lefse analysis (Fig. 2B). Genera *Tapinella* and *Scutellospora* were significantly abundant in NKRD areas; *Cookeina, Yarrowia, Sarea, Ramaria, Cortinarius,* and *Leptoporus* predominated in MKRD areas; and *Sebacina* was significantly abundant in SKRD regions. Changes of genera that were significantly different in KRD regions are shown in Supplementary Figs. 2, 3 and 4. Similar to previous studies^2,3,8,32^, MKRD contained certain unique soil features and microbiome, providing a potential lead for soil and vegetation restoration.

**Table 1:** The correlations of soil fungal richness and diversity with soil properties in karst rocky desertification regions.

	pH	SOM g/kg	TN g/kg	TP g/kg	TK g/kg	AN mg/g	AP mg/g	AK mg/g
Richness	**0.78**	**0.83**	0.56	− 0.19	− 0.10	0.59	− 0.09	**0.68**
Diversity	**0.80**	0.51	0.15	− **0.66**	0.19	0.08	− 0.57	**0.80**

Pearson correlation analysis was used to determine the relationships of richness (Ace) and diversity (Shannon) with soil properties in karst rocky desertification regions. Values in bold are significantly different, *P* < 0.05. Soil organic matter (SOM), total and available nitrogen (TN and AN), total and available phosphorus (TP and AP), and total and available potassium (TK and AK).

**Figure 3:**
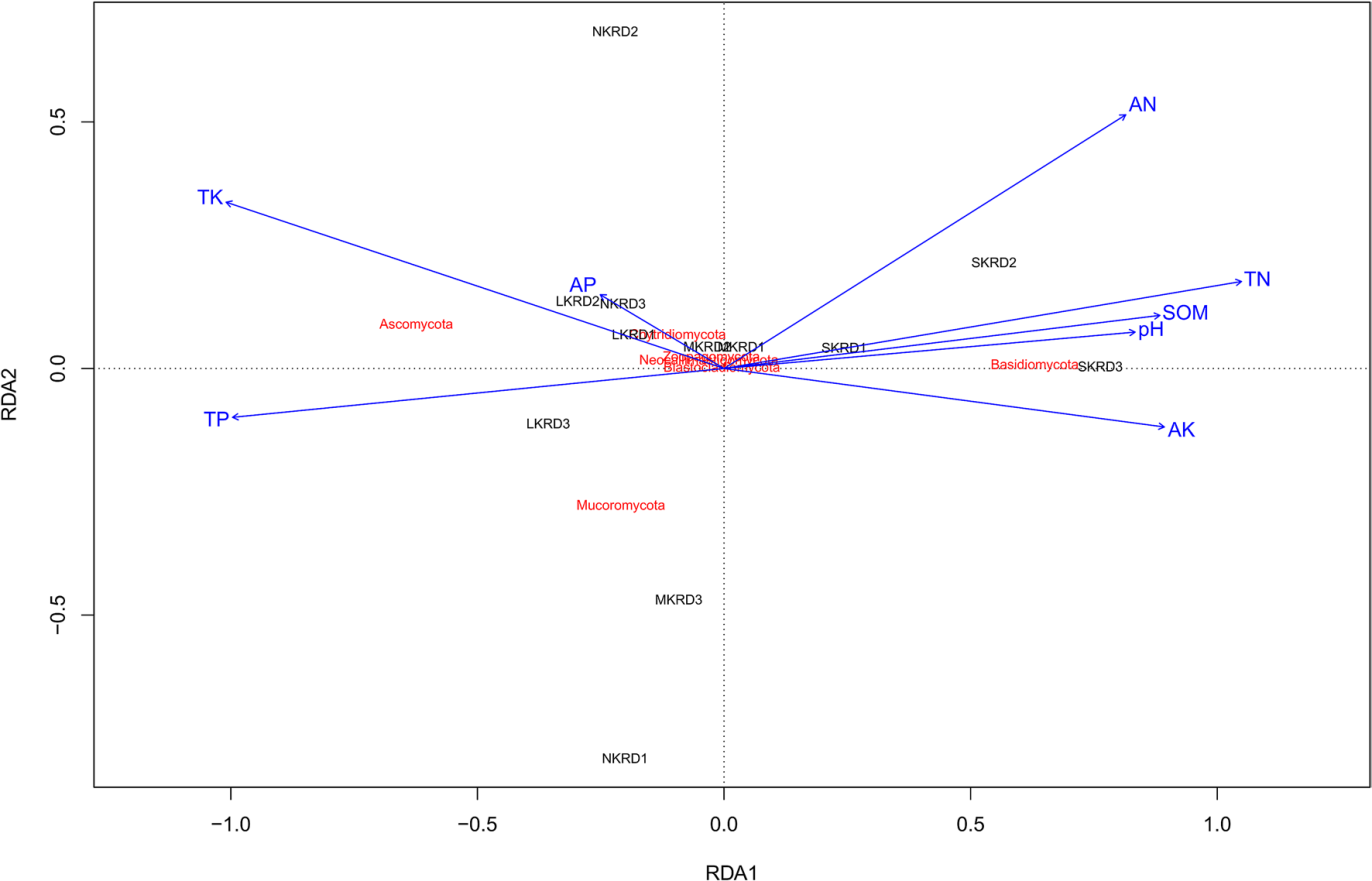
The distance-based redundancy analysis (db-RDA) diagram was used to show distribution of soil fungal communities in karst rocky desertification areas. Study sites are labeled in black No KRD (NKRD), latent KRD (LKRD), moderate KRD (MKRD), and severe KRD (SKRD). Soil properties are labeled in blue. Soil organic matter (SOM), total and available nitrogen (TN and AN), total and available phosphorus (TP and AP), and total and available potassium (TK and AK). Phylum is labeled in red.

**Table 2:** Monte Carlo permutation test was used to determine the relationships of soil fungal community composition with soil properties in karst rocky desertification region.

	RDA1	RDA2	r2	F	P value
TN	0.98	0.12	**0.43**	4.78	**0.005**
AN	0.13	0.02	0.01	1.17	0.53
pH	0.96	0.06	**0.35**	4.31	**0.005**
AK	0.96	− 0.14	0.15	2.13	**0.004**
TK	0.29	− 0.60	0.07	1.17	0.34
AP	− 0.23	0.23	0.06	1.21	0.38
TP	− 0.24	0.15	0.06	1.30	0.29
SOM	0.78	0.12	0.06	1.51	0.43

Values in bold indicate significant associations of soil properties with soil fungal community composition. Soil organic matter (SOM), total and available nitrogen (TN and AN), total and available phosphorus (TP and AP), and total and available potassium (TK and AK).

**Figure 4:**
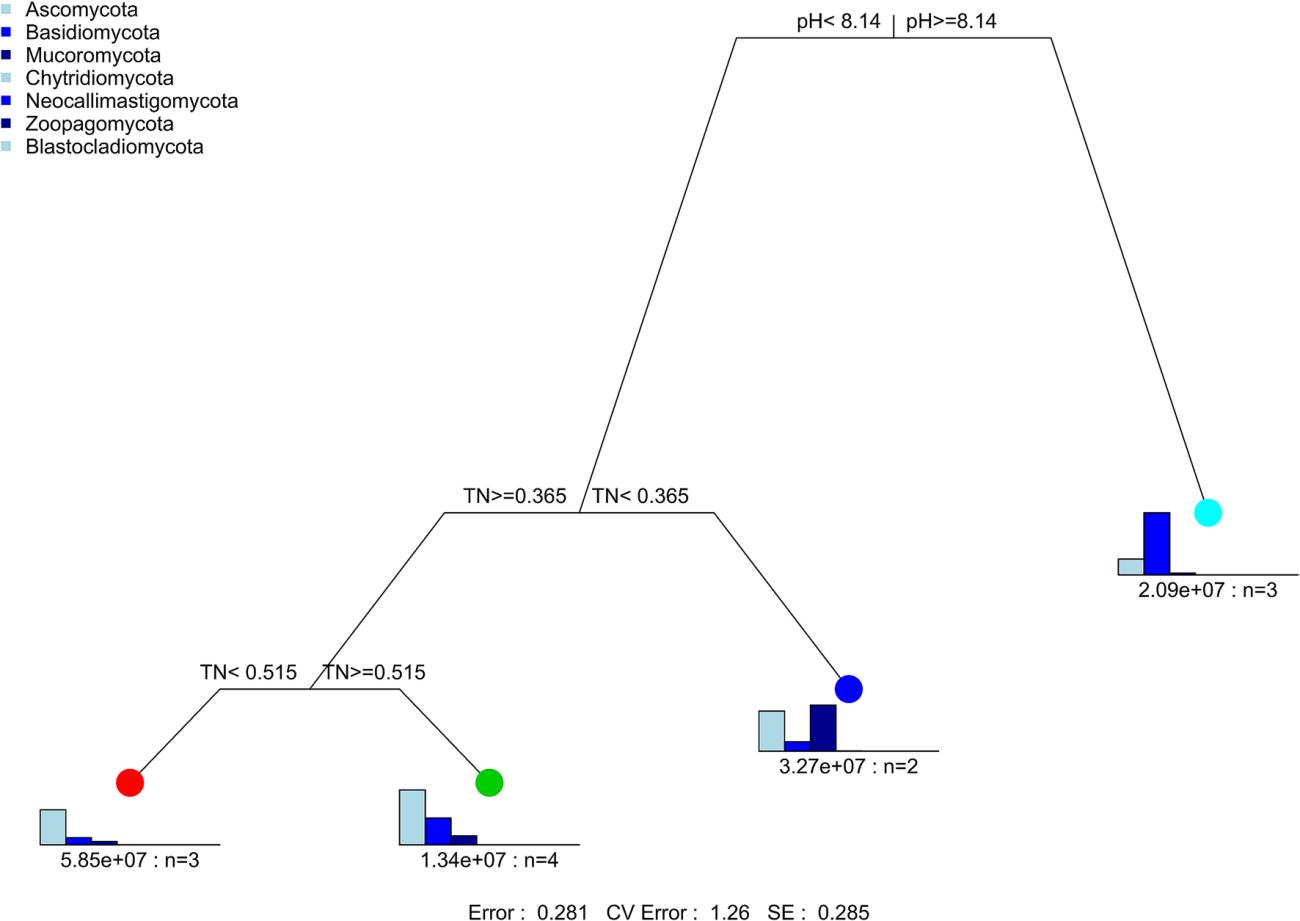
The multivariate regression tree (MRT) was used to reveal the relationship of relative abundance of core phyla with change in soil properties caused by KRD progression. Soil organic matter (SOM) and total phosphorus (TP).

Shannon and Ace indices were calculated to measure diversity and richness in soil fungal communities, respectively (Supplementary Table 2). The overall change in diversity and richness was not significantly different across the KRD gradient. The diversity remained stable along the soil degradation; whereas richness fluctuated and decreased in SKRD compared to that of NKRD. Pearson Correlation analysis indicated that TP had a significantly positive correlation (r2 =  − 0.66, *P* < 0.05) with the richness (Table 1).

**Table 3:** Pearson Correlation analysis was used to determine the relationship of soil properties with soil fungi at phylum level.

Phylum	pH	SOM g/kg	TN g/kg	TP g/kg	TK g/kg	AN mg/g	AP mg/g	AK mg/g
Ascomycota	− 0.49	− 0.57*	− 0.42	0.44	0.43	− 0.41	0.29	− 0.32
Basidiomycota	0.67**	0.71**	0.81***	− 0.78**	− 0.80***	0.63*	− 0.22	0.75**
Mucoromycota	− 0.42	− 0.39	− 0.32	0.30	− 0.04	− 0.52*	0.08	− 0.18
Chytridiomycota	− 0.57*	− 0.43	− 0.34	0.44	0.37	− 0.20	0.19	− 0.49
Neocallimastigomycota	0.12	0.07	0.06	0.31	− 0.03	0.13	− 0.08	0.26
Zoopagomycota	− 0.34	− 0.25	− 0.31	0.34	0.40	− 0.10	0.13	− 0.33
Blastocladiomycota	− 0.55*	− 0.32	− 0.30	0.24	0.026	− 0.17	0.15	− 0.28

Values in bold indicate significant associations. *P < 0.05; **P < 0.01; ***P < 0.0001. Soil organic matter (SOM), total and available nitrogen (TN and AN), total and available phosphorus (TP and AP), and total and available potassium (TK and AK).

**Table 4:** Pearson Correlation analysis was used to determine the relationship of soil properties with soil fungi at genera level.

Genus	pH	SOM g/kg	TN g/kg	TP g/kg	TK g/kg	AN mg/g	AP mg/g	AK mg/g
Sebacina	0.44	0.49	0.59*	− 0.59*	− 0.57*	0.50	− 0.08	0.39
Tomentella	0.34	0.32	0.37	− 0.21	− 0.32	0.33	− 0.24	0.54*
Teratosphaeria	− 0.74**	− 0.74**	− 0.62*	0.49	0.38	− 0.74**	0.58*	− 0.56*
Russula	0.49	0.47	0.42	− 0.48	− 0.37	0.26	− 0.28	0.41
Candida	0.053	− 0.05	0.24	− 0.10	− 0.11	0.03	− 0.02	0.24
Glomus	− 0.47	− 0.41	− 0.15	0.37	− 0.11	− 0.32	0.19	− 0.12
Leccinum	0.47	0.41	0.39	− 0.45	− 0.32	0.18	− 0.27	0.37
Mortierella	0.01	0.01	0.08	− 0.19	− 0.25	0.01	0.09	0.19
Daedalea	0.12	0.26	0.05	− 0.26	− 0.25	0.28	− 0.04	0.31
Cookeina	0.21	0.24	0.32	− 0.33	− 0.36	0.27	0.09	0.46

*P < 0.05; **P < 0.01; ***P < 0.0001. Soil organic matter (SOM), total and available nitrogen (TN and AN), total and available phosphorus (TP and AP), and total and available potassium (TK and AK).

The db-RDA was used to reveal similarity among fungal communities (β-diversity, which was calculated with a unifrac-weighted matrix at phylum level) (Fig. 3). Results showed that soil fungal communities in SKRD areas separated from the rest of communities along the RDA1 axis, indicating a distinguishable difference in community composition in SKRD compared to those of other KRD areas. Soil microbiome in N- and L-KRD regions were indistinguishable. The fungal community in MKRD, however, was further separated from those of in N- and L-KRD along RDA2 axis, suggesting a gradual change in soil microbiome associated with KRD progression.

A Monte Carlo permutation test at 999 permutations was used to elucidate the relationship of soil properties with fungal community composition (Table 2). Overall, the set of eight soil properties explained 81.3% of the variances, with RDA1 axis explaining 73.3% of the variance and axis 2 explaining 7.9%. Soil TN (r2 = 0.43, *P* = 0.005) was the primary factor that significantly associated with soil microbiome in KRD regions, followed by pH (r2 = 0.35, *P* = 0.0.05).

The MRT analysis (Fig. 4) was used to interpret the relationship of relative abundance of core phylum with changes in soil properties during KRD progression. Results indicated that Basidiomycota were the most abundant in soils with pH higher than 8.16. This pH condition was only found in NKRD soils. Ascomycota and Mucoromycota predominated in soils with TN value lower than 0.37, which only existed in N- and L-KRD areas.

**Figure 5:**
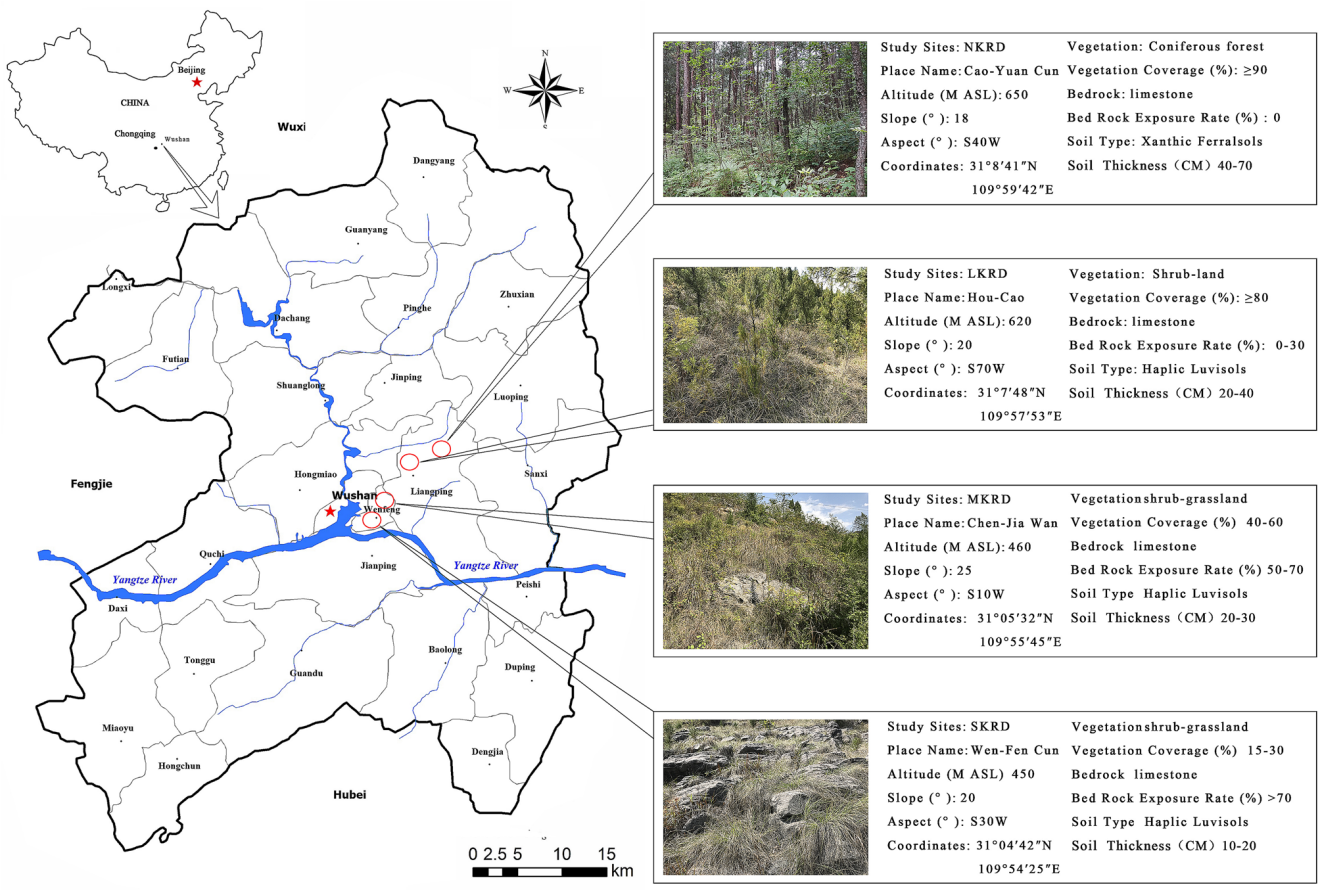
Study sites overview. Geographic features, soil type, bedrock exposure rate, and vegetation coverage are summarized in the figure. Classification of KRD is based on vegetation coverage, soil depth, and bedrock exposure rates. No KRD (NKRD), latent KRD (LKRD), moderate KRD (MKRD), and severe KRD (SKRD). Figure 1 was generated by the authors using mapinfo (v. 12.0.2) and Adobe Photoshop (v.13.0).

The relationship of soil properties with the core phyla and genera was further assessed using Pearson Correlation analysis (Table 3 and 4). Basidiomycota was the most sensitive phylum responding to changes in soil properties; it had significant correlations to all the soil properties except for AP. Specifically, Basidiomycota had significantly positive correlation to pH (r2 = 0.67), SOM (r2 = 0.71), TN (r2 = 0.81), AN (r2 = 0.63), and AK (r2 = 0.75) TN (r2 = 0.69); and significantly negative correlation to TP (r2 =  − 0.78) and TK (r2 = 0.80). Soil pH had a negative correlation to Chytridiomycota (r2 =  − 0.57) and Blastocladiomycota (r2 =  − 0.55). Change in SOM negatively correlated to the relative abundance of Ascomycota (r2 =  − 0.57); whereas AN negatively associated with Mucoromycota (r2 =  − 0.52). *Teratosphaeria* was the most sensitive genera to changes of soil properties, followed by *Sebacina* and *Tomentella*. Specifically, *Teratosphaeria* was negatively correlated with soil pH, SOM, and TN, yet positively correlated with AP. *Sebacina* was negatively correlated with TP and TK, positively correlated with TN. *Tomentella* was positively correlated with AK.

## Discussion

The progression of KRD has a large impact on the local ecosystem, agriculture, and economy; as well as on the global ecosystem. Most of the studies focus on interactions of KRD with macroorganisms (e.g., plants, animals, human activities, etc.). The dynamics between KRD and microorganisms are largely unknown, especially on the soil fungal community. This study aims to survey the fungal community in different KRD progressing stages, characterize the change in the fungal community, and attempt to understand the interactions between changes in soil properties and fungal community.

Unlike vegetation, this study found that the overall richness and diversity of soil fungal communities did not significantly change along with progressive land degradation in the karst region. Similar findings were reported in previous studies, where soil microbiome was abundant and diverse in extreme environments, such as high Arctic area, high altitude mountains, and soil of low fertility^20,22,31,33^. Specifically, results showed that fungal communities in NKRD and SKRD were distinguishably different. This finding is congruent to the previous studies^10,34^. The richness of the fungal community in SKRD was slightly lower than that in NKRD. The change in soil fungal community along with the KRD progression implies a mutual impairment between deteriorated ecosystems and simplified microbial communities. Indeed, previous studies demonstrated that the change in soil microbial community, as well as the loss of soil biodiversity, endangered the functionality and sustainability of the ecosystem^34–37^. Moreover, changes in soil fungal communities impact the nutrient cycling, especially the litter decomposition and nitrogen reincorporation, which liberate nitrogen back to above-ground communities. The disintegrated nutrient cycling and soil structure potentially explain the degraded vegetation communities found in SKRD areas^38^.

One of the most intriguing results in this study was that phylum Ascomycota claimed predominance in NKRD, while Basidiomycota increased in desertified land and peaked in SKRD. The ratio of Ascomycota/Basidiomycota significantly decreased along with KRD progression. The same trend was also reported in previous studies, suggesting that the nutrient availability and fungal activities, especially fungal decomposition are two key elements for soil restoration^27,39^.

Genus *Sebacina* (Basidiomycota) was overall the most abundant genus found in this study. Moreover, *Sebacina* were significantly predominant in SKRD areas, indicating the strong colonization ability in the terrestrial environment. Previous studies suggested that genus *Sebacina* had high decomposition potentials; therefore, was more active in soils with relatively high SOM, C, and N contents^40,41^. Although SKRD had the least vegetation coverage, richness, and diversity, the soils in SKRD had SOM of rather high concentration. This might be due to the high dissolution rate of limestone and accumulation of recalcitrant SOM in SKRD^42^. The microbial composition and availability of fresh carbon cooperatively affect SOM decomposition. This finding was congruent to previous studies, revealing the relatively widespread of *Sebacina* and their potentials to assist nutrient cycling in degraded soils. Interestingly, besides the decomposition functions in the terrestrial soils, *Sebacina* also contain species, e.g., *S. vermifera,* which are unique root symbionts promoting plant performance^13,43,44^. These results indicate that *Sebacina* has the potential to release more accessible nutrients to plants and to boost the plants growth. Altogether, results suggest that *Sebacina* might have the potential to contribute to vegetation restoration in KRD areas.

Mucoromycota, Chytridiomycota, and Neocallimastigomycota were relatively abundant in NKRD. All of these phyla are known for their plant-derived cellulose degrading and litter decomposition functions^27^. NKRD had the highest vegetation coverage, thereby maintaining the thickest humus layer. The relatively high prevalence of phyla in NKRD suggested a close relationship of fungal activity with overall soil quality and condition.

Results suggested that soil TN and pH were the most influential environmental factors associated with fungal community composition. Noticeably, soil TN and pH were significantly higher in SKRD than those in NKRD areas. The strong correlation of fungal community with soil TN and pH was also reported in previous studies^45–47^. The deteriorated soil conditions (e.g., low water retention, low structural integrity, etc.) in SKRD led to the significant accumulation of recalcitrant soil nutrients, which had rather high nutrient concentration in measurement but were inaccessible to vegetation. It was demonstrated that fungi, triggered by fresh carbon, could decompose recalcitrant SOM and nitrogen, known as the priming effect^47^. *Sebacina* (Basidiomycota), which predominated in SKRD, had a significantly positive correlation with TN and negative correlations with TK and TP. It was possible that the soil condition in SKRD (e.g., high recalcitrant soil nutrients, lack of soluble nutrients and water retention) favored *Sebacina* over other genera. More specifically, the high TN might positively select *Sebacina* for SOM decomposition. Such mechanism is similar to r-strategist microbes^48^. *Teratosphaeria* had a significantly positive correlation with AP and negative correlations with pH, SOM, TN, AN, and AK. Fungi like *Teratosphaeria* decompose cellulose in the long term (several decades) and release fresh carbon, which might trigger the priming effect of *Sebacina* and release soil nutrients.

Besides TN, soil pH was the second most influential factor associated with fungal community composition. Except for NKRD area (pH 5–6), soil in KRD region is mainly alkaline (pH > 7.0). Genera *Teratosphaeria* and *Tomentella* predominated (> 50%) in NKRD. Genus *Tomentella* is considered as a short-distance exploration that prefers mild moist, neutral pH, and fertile soil^49^. In comparison, *Russula* (phylum Basidiomycota, Order Agaricales) significantly predominated in SKRD, indicating their strong colonization abilities in alkaline soil. Interestingly, this study found members from the same class (Agaricomycetes) had a rather wide range of pH preference. Such diversity suggested fungi had various responses to the surroundings. It also indicated that soil pH was not the only environmental factor impacting the distribution of fungal community. Beside the high alkaline tolerance, class Agaricomycetes has a large group of fungi that effectively digest complex and recalcitrant carbon like lignin, waxes, and tannins^27^. Altogether, it may explain the prevalence of *Russula* in harsh environments such as SKRD regions.

Sebacinales was the most abundant order and significantly predominated in SKRD. Sebacinales also have a broad soil pH (pH 5.5–8.5) adapting range^50^, which enables them to have higher competitiveness in the fungal community. Such features might explain the prevalence of Sebacinales especially in soil with high pH, observed in this and previous studies^23,46,51^.

The fungi-pH interaction found in this study concurs with previous reports^31,52^. Many studies demonstrated that soil pH had both direct and indirect influence on fungal communities. Fungi show various colonization preferences to soil pH. For example, lichen-forming fungi predominate in soil with pH higher than 8.0, while non-lichen forming ones are prevalent in pH lower than 8.0^53,54^. Furthermore, fungi with broader soil pH range colonize more aggressively than those with narrower pH range. Soil pH also modifies fungal community establishment indirectly via interactions with plants. Soil pH has a strong impact on the vegetation community composition; while roots-fungi interaction, in turn, promotes certain types of plants to grow^55–57^. Other soil properties (e.g. SOM and N) were reported to have significant effects on fungal communities^55,58,59^. Results found in this study concur with previous studies.

In summary, this study characterized soil fungal communities in KRD regions and revealed the change in soil fungal communities along with KRD progression. Furthermore, this study demonstrated that soil TN and pH were the main factors associated with the fungal community in KRD areas. Moreover, this study found the Ascomycota/Basidiomycota ratio significantly decreased along with the KRD progression, which might be used as an indicator to predict KRD progression. This is one of the first reported cases profiling the fungal community in KRD regions using the high-throughput sequencing technology. Some further investigations attempting to understand the interactions between soil bacterial and fungal communities, as well as seasonal changes of soil microbiome are ongoing as continuous studies, in order to comprehensively understand microbiome ecology in KRD regions.

## Methods

### Study area

The study sites are located in Wushang County, northeast of Chongqing, China (Fig. 5). Detailed description is reported in previous study^3^. Briefly, the study sites were classified into non-KRD (NKRD), latent KRD (LKRD), moderate KRD (MKRD), and severe KRD (SKRD) based on the bare rock exposure rate, vegetation coverage rate, and bedrock exposure. Haplic Luvisols is the main soil type in L-, M-, and S-KRD regions while Xanthic Ferralsols is the main soil type in the NKRD region. The soil type was identified by following guidelines of Food and Agriculture Organization of the United Nations (FAO)^60^. Other variances such as mean annual air temperature (18.4 ℃) and mean annual precipitation (1049.3 mm) are consistent in all the sites.

Quadrats (20 m × 20 m) were assigned for this study. A total of 12 sampling quadrats, three replicates for each KRD stage were selected. Within each quadrat, three subsampling quadrats (10 m × 10 m) were uniformly selected, with the purpose of comprehensively sampling communities.

### Soil sampling

The soil samples were collected in KRD areas with little human interference. All the sampling tools were autoclaved. The sampling method followed the description reported in a previous study^32^. Briefly, in each subsampling quadrant, three soil samples (5 g) were randomly and uniformly collected at the top of the soil layer (0–10 cm) using a soil push probe. No plant residues were included in the soil sample. Soil samples were stored in 50 mL sterile tubes on ice. Three samples from one subsampling quadrant were pooled into the same 50 mL tubes (15 g) in order to comprehensively represent soil microbiome in a quadrant. All pooled samples from sampling quadrats were labeled as NKRD_1, NKRD_2, NKRD_3, LKRD_1, LKRD_2, LKRD_3, MKRD_1, MKRD_2, MKRD_3, SKRD_1, SKRD_2, and SKRD_3. Once arrived at the lab, all the soil samples were immediately mixed in a Large Capacity Mixer (Glas-Col, Terre Haute, IN, USA) at 1600 rpm for 30 min at 4 °C. Two sets of soil subsamples (2 g) were aliquoted under a sterile environment. One set of samples was used for soil property measurement. The other set was for sequencing.

### Soil properties measurement

Soil samples were sent to the Key Laboratory of Eco-environments in Three Gorges Reservoir Region for property measurement. Soil pH, SOM, TN, AN, TP, AP, TK, and AK were measured. Detailed protocols were reported in a previous study^3^. Briefly, soil pH was diluted to 1:2.5 (w/v) and measured with a pH meter (FE20, Mettler-Toledo Instruments, China). The potassium dichromate oxidation was used to measure the soil organic carbon (SOC)^61^and SOM was calculated by multiplying SOC values by 1.724^62^. LAQUAtwin ion meter kits (Horiba Instruments, Singapore) were used to measure TN, AN, TK, AK, TP, and AP. All the soil properties were measured in biological and technical triplicate.

### DNA isolation

The soil DNA was isolated by using MoBio PowerSoil DNA extraction kit (MoBio Laboratories, Carlsbad, CA, USA) following the manufacturer's instructions with a few alterations. Briefly, 0.5 g subsampled soil was mixed with the beads and lysing buffer. An additional 10 min incubation time was conducted at 65 °C, then the bead beating was performed for 2 min^25^. A 0.8% (wt/vol) low melting point agarose gel was used to evaluate the DNA integrity. DNA was purified by using the agarose gel DNA purification kit (TaKaRa Bio USA, Inc., Mountain View, CA, USA). A NanoDrop One spectrophotometer was used to measure the DNA quantity (NanoDrop Technologies, Wilmington, DE, USA).

### PCR amplification and ITS1 region sequencing

The template contained 50 ng DNA for PCR amplification. The ITS1 region was amplified using the primer set: ITS1F (CTTGGTCATTTAGAGGAAGTAA) and ITS2R (GCTGCGTTCTTCATCGATGC)^63^. The amplification conditions were: 94 °C for 3 min, followed by 28 cycles of 94 °C for 30 s, 57 °C for 40 s, and 72 °C for 60 s, and finally 72 °C for 5 min. Phusion High-Fidelity PCR Master Mix (New England Biolabs, Ipswich, MA, USA) was used for the reaction. PCR products were evaluated by using a 2% electrophoresis agarose gel. Qiagen Gel Extraction Kit (Qiagen, Hilden, Germany) was used to purify the PCR products.

The library was indexed and purified before normalization. Qubit 3.0 Fluorometer (Thermo Scientific, Waltham, MA, USA) was used to measure the DNA concentration.Agilent Bioanalyzer 2100 system (Agilent, Santa Clara, CA, USA) was used to determine the library size. The Illumina HiSeq2500 sequencer (Illumina, San Diego, CA, USA) was used to sequence the pooled library.

### Bioinformatic analysis

The data mining was performed by using Mothur (v. 1.40)^64^. Briefly, paired-end reads were assembled and assigned to each sample based on their unique barcode by using ‘make.contigs’ command. Sequences of each sample were then truncated by removing the barcode and primer sequences. Sequences were excluded if they were longer than 324 bp, or contained longer than 8 homopolymers, or contained undermined base, or did not align to the ITS1 region, with ‘screen.seqs’ command, and criteria as ‘maxambig = 0, maxhomop = 8, and maxlength = 324″. Qualified contigs were further processed with commands, such as ‘trim.seqs’ and ‘pre.cluster’. Uchime was used to remove chimeric sequences with command ‘chimera.uchime’^65^. Command ‘classify.seqs’ was used to classify sequences by using the Bayesian classifier. The UNITE database^66^ was used as the reference. The alignment was performed with the parameters ‘method = knn, search = blast, match = 2, mismatch =  − 2, gapopen =  − 2, gapextend =  − 1, numwanted = 1′. Command ‘remove.lineage’ with criteria ‘taxon = Chloroplast-Mitochondria-unknown-Archaea-Eukaryota’ was used to exclude information from Archaea, chloroplasts, and mitochondria. Qualified sequences were classified into operational taxonomic units (OTUs) based on at least 97% similarity (OTU_0.03_) by using command ‘cluster.split’. All the sequences of OTU_0.03_ were assigned into taxonomic groups at the bootstrap threshold of 80% by using command ‘classify.otu’. Command ‘sub.sample’ was used to randomly normalize all the samples with the least amount of sequences in all the samples, in order to avoid sequencing bias. Alpha diversity was calculated by using the command ‘summary.single’. The beta diversity was measured by using unifrac-based metrics generated with command ‘unifrac.weighted’^67^. Raw sequencing data was submitted to Sequence Read Archive (SRA) in NCBI, access number PRJNA623298.

### Statistical analysis

R 3.3.2 statistical software^68^ was used to perform One-way Analyses of variance (One-way ANOVA) and Pearson correlation analyses. The significant changes in soil properties caused by KRD progression was determined by using Tukey's honest significant difference (HSD) test. The change was labeled as significant when *P* value < 0.05. Pearson correlation analysis was used to determine the relationship of soil properties with fungi at phylum and genera level. The correlations were considered significantly when the *P* < 0.05.

The weighted unifrac distance was used to determine soil fungal community structural similarity^67^. Communities with similar compositions closely clustered, and vice versa. The multivariate regression tree (MRT) was built by using the mvpart package in R^69^. The MRT tree was used to characterize the relationship of relative abundance of core phyla with changes in soil properties during the KRD progression.

Distance-based redundancy analysis (db-RDA) was performed by using the vegan package in R^70^. The db-RDA was used for direct gradient analysis. Two ordination exes (RDA1 and RDA2) showed the scattering of constraints and accumulated explanatory variables. The ordinations axes (RDA1 and RDA2) were constructed by constraining an entire set of explanatory variables (eight soil properties). The impact of soil properties on fungal community composition was assessed by using the Mantel test at 999 permutations. Impact of soil properties on the microbiome community were considered significant when *P* < 0.05.

Linear discriminant analysis (LDA) Effect Size (LEfSe) was used to determine the change in relative abundance of soil fungi responding to KRD progression^71^. The non-parametric factorial Kruskal–Wallis (KW) sum-rank test was first used to determine fungi with significant abundant differences between KRD stages (*P* < 0.05). The unpaired Wilcoxon rank-sum test was used to compare the significance of abundant difference among taxa under impact of soil desertification (*P* < 0.05). Linear Discriminant Analysis was applied to calculate the effective size of abundant differences. The LAD scores were normalized by log10.

## Supplementary Information

Supplementary Legends.

Supplementary Figure 1.

Supplementary Figure 2.

Supplementary Figure 3.

Supplementary Figure 4.

Supplementary Table 1.

Supplementary Table 2.
