# Supplementary material for: Total nitrogen is the main soil property associated with soil fungal community in karst rocky desertification regions in southwest China
Source: Sci Rep. 2021 May 24;11:10809. doi: 10.1038/s41598-021-89448-1 (PMC8144601; doi:10.1038/s41598-021-89448-1)
Supplement: Supplementary file 1 — Supplementary Legends. [file 41598_2021_89448_MOESM1_ESM.docx]

**Supplementary FIGURE 1. The Linear discriminant analysis (LDA) Effect Size (LEfSe) analysis was used to identify phyla that were significantly different in each karst rocky desertification region.** Relative abundance is significant when P < 0.05, logarithmic LDA score ≥ 2.0. No KRD (NKRD), Latent KRD (LKRD), Medium KRD (MKRD), and severe KRD (SKRD).

**Supplementary FIGURE 2-4. The Linear discriminant analysis (LDA) Effect Size (LEfSe) analysis was used to identify genera that were significantly different in each karst rocky desertification region.** Relative abundance is significant when P < 0.05, logarithmic LDA score ≥ 2.0. No KRD (NKRD), Latent KRD (LKRD), Medium KRD (MKRD), and severe KRD (SKRD).
