# Supplementary figures and images for: Total nitrogen is the main soil property associated with soil fungal community in karst rocky desertification regions in southwest China
Source: Sci Rep. 2021 May 24;11:10809. doi: 10.1038/s41598-021-89448-1 (PMC8144601; doi:10.1038/s41598-021-89448-1)

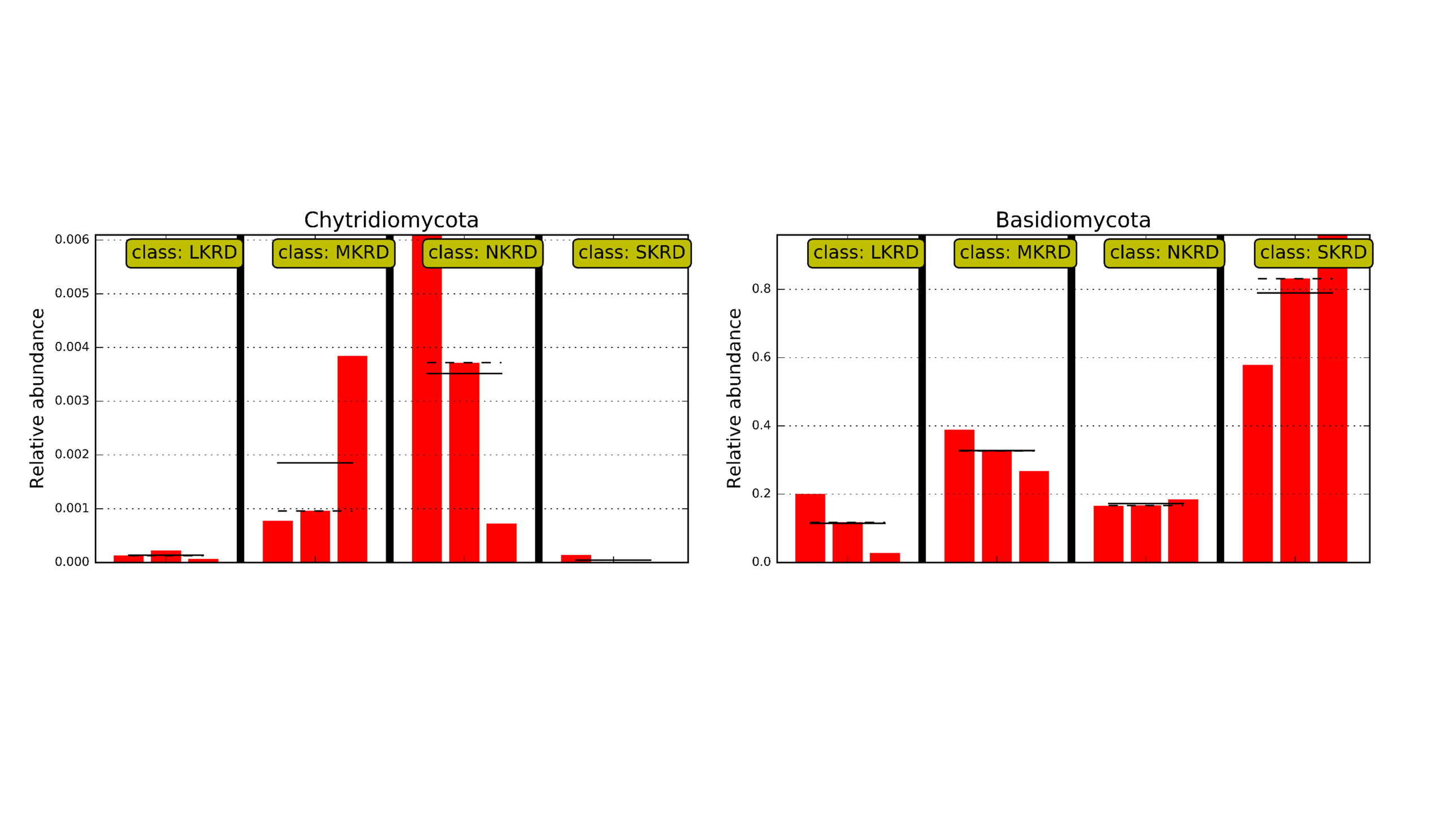

Supplement: Supplementary file 2 — Supplementary Figure 1. [file 41598_2021_89448_MOESM2_ESM.tif]

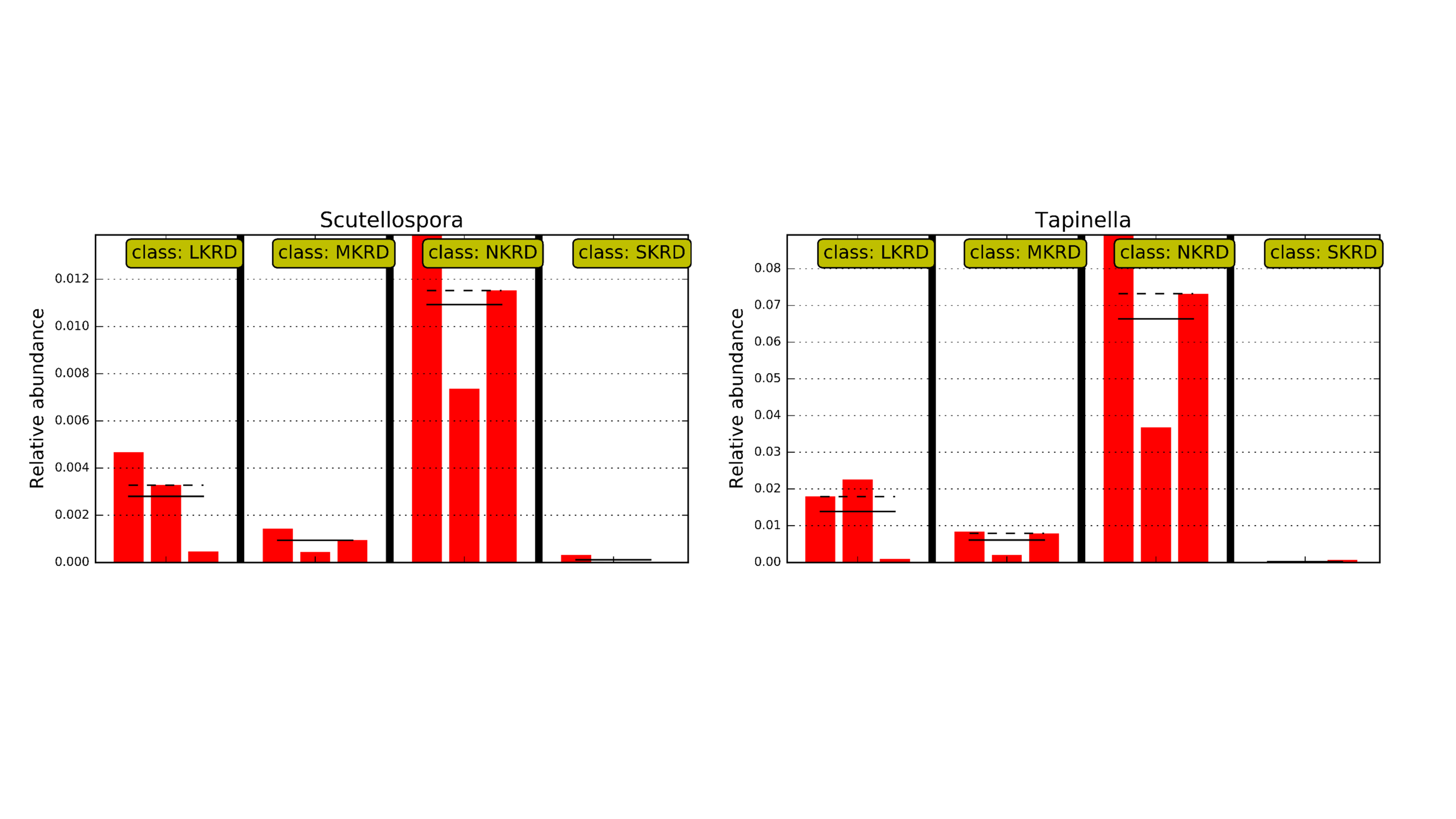

Supplement: Supplementary file 3 — Supplementary Figure 2. [file 41598_2021_89448_MOESM3_ESM.tif]

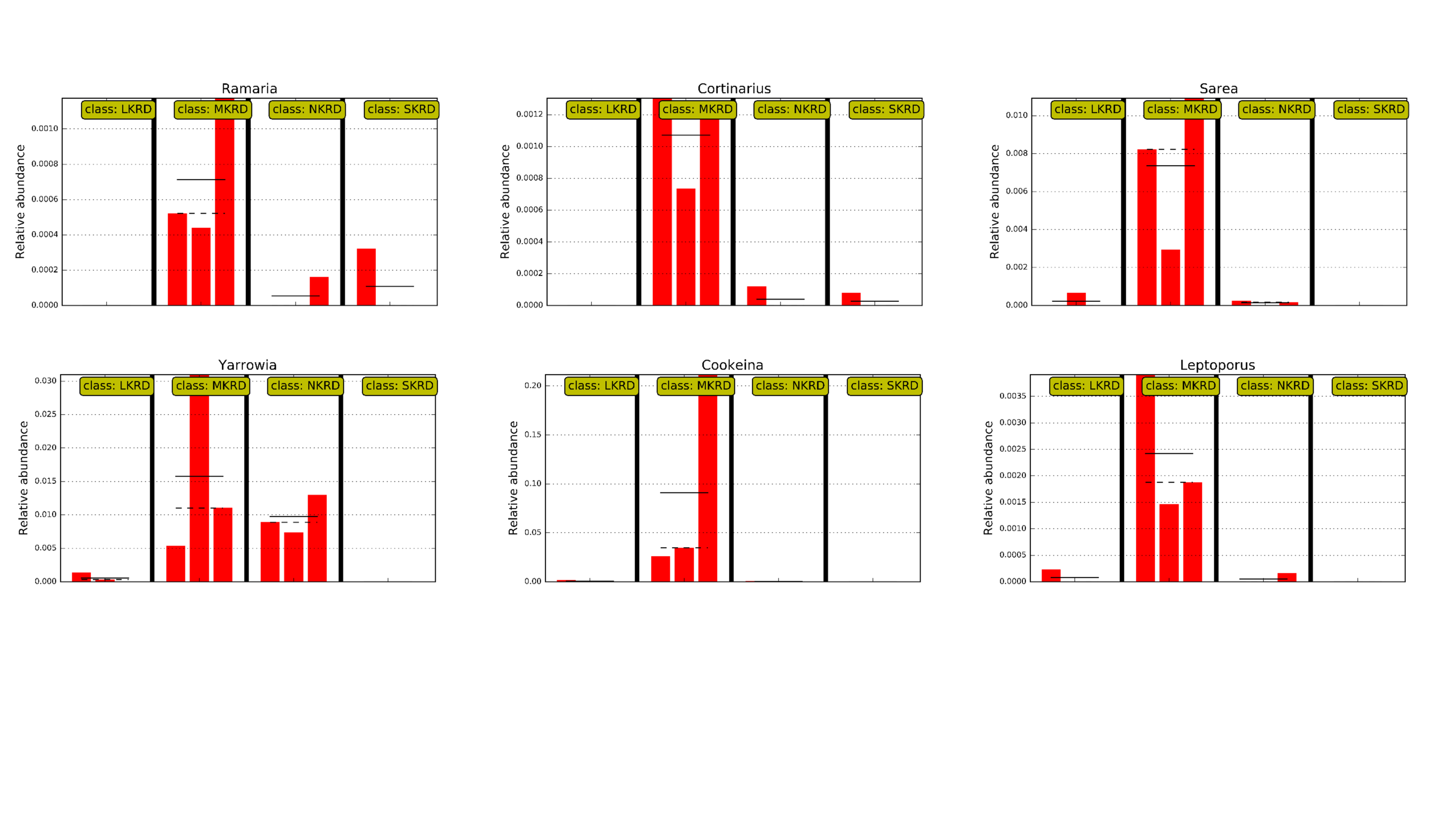

Supplement: Supplementary file 4 — Supplementary Figure 3. [file 41598_2021_89448_MOESM4_ESM.tif]

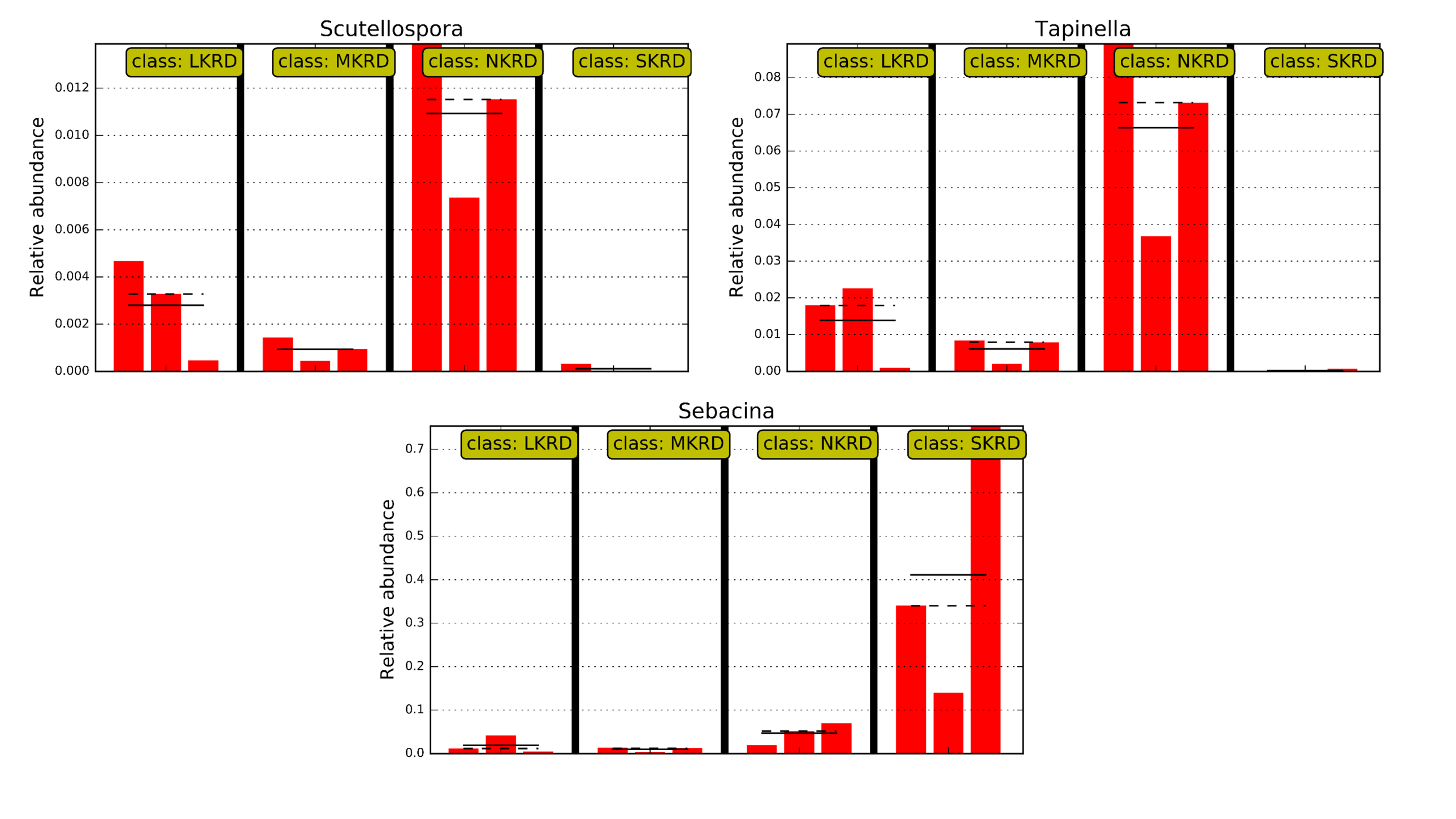

Supplement: Supplementary file 5 — Supplementary Figure 4. [file 41598_2021_89448_MOESM5_ESM.tif]
