# Supplementary material for: Total nitrogen is the main soil property associated with soil fungal community in karst rocky desertification regions in southwest China
Source: Sci Rep. 2021 May 24;11:10809. doi: 10.1038/s41598-021-89448-1 (PMC8144601; doi:10.1038/s41598-021-89448-1)
Supplement: Supplementary file 7 — Supplementary Table 2. [file 41598_2021_89448_MOESM7_ESM.docx]

**Supplementary Table 2.** Soil fungal richness and diversity in karst rocky desertification region.

|  | **Richness (Ace)** | **Diversity (Shannon)** |
| --- | --- | --- |
| **NKRD1** | 5.77 | 17726.07 |
| **NKRD2** | 5.16 | 13471.67 |
| **NKRD3** | 5.83 | 9003.83 |
| **LKRD1** | 5.41 | 15809.55 |
| **LKRD2** | 5.42 | 6188.61 |
| **LKRD3** | 5.86 | 3907.88 |
| **MKRD1** | 5.43 | 8359.38 |
| **MKRD2** | 4.85 | 27753.00 |
| **MKRD3** | 4.59 | 10425.93 |
| **SKRD1** | 5.87 | 8410.27 |
| **SKRD2** | 5.48 | 4964.65 |
| **SKRD3** | 5.97 | 5133.90 |

No KRD (NKRD), Latent KRD (LKRD), Medium KRD (MKRD), and severe KRD (SKRD). Soil organic matter (SOM), total and available nitrogen (TN and AN), total and available phosphorus (TP and AP), and total and available potassium (TK and AK).
